# Postoperative Acute Intracranial Hemorrhage and Venous Thromboembolism in Patients with Brain Metastases Receiving Acetylsalicylic Acid Perioperatively

**DOI:** 10.3390/curroncol31080343

**Published:** 2024-08-10

**Authors:** Nikolay Tonchev, Anatoli Pinchuk, Claudia A. Dumitru, Belal Neyazi, Vanessa Magdalena Swiatek, Klaus Peter Stein, Ibrahim Erol Sandalcioglu, Ali Rashidi

**Affiliations:** Department of Neurosurgery, Otto-von-Guericke-University Magdeburg, Leipziger Straße 44, 39120 Magdeburg, Germany; nikolay.tonchev@med.ovgu.de (N.T.); anatoli.pinchuk@med.ovgu.de (A.P.); belal.neyazi@med.ovgu.de (B.N.); vanessa.swiatek@med.ovgu.de (V.M.S.); klaus-peter.stein@med.ovgu.de (K.P.S.); erol.sandalcioglu@med.ovgu.de (I.E.S.)

**Keywords:** ASA, metastases, craniotomy, hemorrhage

## Abstract

Cranial operations are associated with a high risk of postoperative intracranial hemorrhage (pICH) and venous thromboembolic events, along with increased mortality and morbidity. With the use of acetylsalicylic acid (ASA) for prophylaxis becoming more prevalent, the risk of bleeding when ASA is administered preoperatively is unknown, as are the effects of discontinuation upon the occurrence of thromboembolic events, especially in societies with aging demographics. To address these questions, a retrospective analysis was performed using medical records and radiological images of 1862 patients subjected to brain tumor surgery over a decade in our department. The risk of pICH was compared in patients with metastases receiving ASA treatment versus patients not receiving ASA treatment. The occurrence of venous thromboembolic events after surgery was also evaluated. The study group consisted of 365 patients with different types of brain metastases. In total, 20 patients suffered pICH and 7 of these were associated with clinical neurological deterioration postoperatively. Of the 58 patients who took ASA preoperatively, 2 patients experienced pICH, compared with 5 patients in the non-ASA impact group (*p* = 0.120). Patients who took ASA were not at significantly higher risk of pICH and therefore a worse outcome compared to the group without ASA. Therefore, these data suggest that in patients at high cardiovascular risk, ASA can be safely continued during elective brain tumor surgery.

## 1. Introduction

Brain metastases represent the most common type of intracranial neoplasm and are observed in 10–30% of all patients suffering from systemic cancer [1,2]. It has been postulated that certain brain tumors, notably meningiomas and glioblastomas, induce a systemic coagulation activation that sharply decreases postoperatively, consequently elevating the risk of rebleeding after surgical resection [3]. This phenomenon is particularly important in the context of brain metastases, originating from melanoma or renal cell carcinoma, which are known for their elevated propensity for spontaneous ICH [4].

The perilous repercussions of hemorrhagic complications stemming from craniotomy procedures are extensively documented, underscoring the potential lethality of such outcomes [5,6]. Complicating matters further, a substantial number of patients, especially among the elderly demographic, routinely incorporate antiplatelet agents such as acetylsalicylic acid (ASA) into their regimen for primary or secondary prophylaxis against cardiovascular disease [7,8]. The administration of ASA introduces a therapeutic dilemma in neurosurgical patients, where the already escalated risk of hemorrhage in surgically removed metastases may be exacerbated by the impact of ASA. However, the possible associations between these two risk factors have not been explored in a systematic manner. Encouragingly, several neurosurgical trials focused on cranial and spinal surgeries have not reported any discernible escalation in the risk of postoperative hemorrhage with ASA [9,10,11].

Postoperatively, patients are given anticoagulants for thromboprophylaxis. The cautious attitude of many neurosurgeons towards prophylactic anticoagulants is due to the specter of postoperative intracranial hemorrhage (pICH), a dangerous scenario with well-documented catastrophic consequences [12,13,14,15,16,17,18,19]. During the course of their disease, 20–30% of patients with high-grade intracranial tumors develop venous thromboembolism (VTE) [4,15,20]. The genesis of VTE in brain tumor patients, attributed to increased local synthesis of tissue factors, compromised postoperative mobility, hemiparesis, and genetic predisposition, unfolds as a complex tapestry [7,12,21,22]. The CLOT trial, which analyzed the efficacy of low-molecular-weight heparin in malignancies, showed a nuanced landscape, with only 2 of 27 brain tumor patients encountering pICH complications [23]. However, a meta-analysis by Zwicker et al. suggested a collective apprehension and noteworthy inclination towards an augmented risk of hemorrhage despite individual studies deeming anticoagulant medication safe [24]. Thus, the combination of pICH tendency of some brain tumors along with the administration of ASA preoperatively and anticoagulants after surgery make a statement regarding postoperative hemorrhage extremely difficult.

To address these questions, we performed a comprehensive evaluation of both postoperative hemorrhage and VTE risks. The focus was particularly directed towards predisposed patients undergoing diverse cranial metastatic surgeries and therefore contingent upon the unique characteristics of the tumor. At the same time, the influence of ASA administration was considered to shed light on the intricate interplay of multiple factors within these therapeutic challenges.

## 2. Materials and Methods

**Figure 1:**
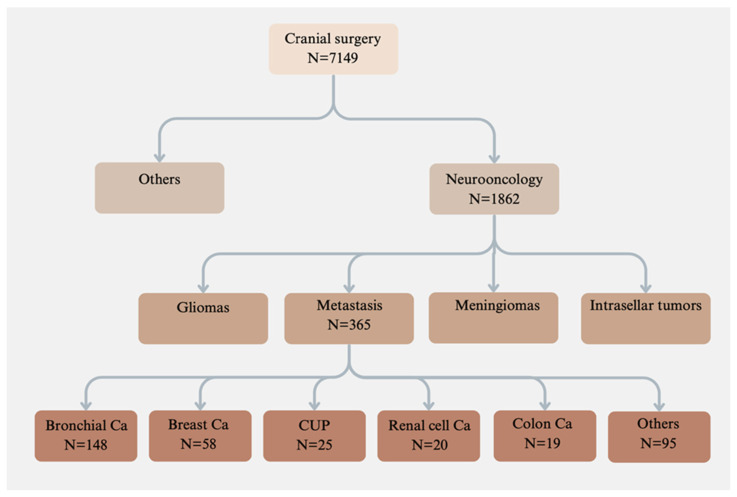
Types of tumors included in the study. All oncological surgical patients in our hospital were examined over a 10-year period. A total of 365 intracranial metastases required surgery. Various metastasis types are shown. Ca, cancer; CUP, cancer of unknown origin.

A thorough retrospective analysis was conducted on the medical records and radiological images of patients who underwent cranial operations at our department between 2008 and 2018. A review of 1862 tumor operations performed during this decade was conducted, with a specific focus on 365 patients who underwent surgery for intracranial metastases (Figure 1).

The following patient parameters were examined from medical records: age, sex, blood type, body mass index (BMI), perioperative administration of ASA, hypertension, diabetes, history of smoking, cardiovascular disease, renal disease, chronic inflammation, repeated surgery (secondary meningioma surgery), laboratory parameters, length of hospital stay, surgical procedure, subtypes of metastases, duration of surgery, blood loss, and postoperative complications.

Complications during hospitalization were classified according to the scheme proposed by Ibanez et al.: grade I denoted non-life-threatening abnormalities within the typical postoperative course that was manageable without invasive procedures; grade II complications necessitated invasive interventions including surgical, endoscopic, or endovascular procedures; grade III complications represented life-threatening adverse events mandating treatment in an intensive care unit and is further divided into IIIa for single organ dysfunction complications and IIIb for multiple organ dysfunction complications; and grade IV complications encompassed fatalities resulting from complications.

Pre- and postoperative contrast-enhanced magnetic resonance imaging was undertaken, contingent upon the absence of contraindications. Steroids were administered preoperatively in the presence of tumor edema or space-occupying effects. Surgeons had access to intraoperative ultrasound and electrophysiologic monitoring, with frameless neuronavigation available as needed.

Tumor characteristics were documented including metastatic subgroups, tumor size, localization of the tumor, recurrent surgery, and degree of resection. Operative parameters such as blood loss during surgery, duration of surgery, extent of resection, and other pertinent characteristics were also recorded. The Karnofsky Performance Scale (KPS) before surgery and the Glasgow Outcome Scale (GOS) after surgery provided a standardized assessment of patient status.

The exclusion criteria comprised patients under the age of 18, pregnant individuals, and those receiving alternative antiplatelet agents like clopidogrel and/or anticoagulants such as Marcumar^®^.

The Fisher’s exact test was used to statistically assess the potential influence of ASA on postoperative hemorrhage. Patients were categorized into two groups:

- No ASA impact, comprising patients without a history of ASA use and/or those who discontinued ASA intake (>7 days before surgery).
- ASA impact, encompassing patients who continued ASA intake (≤7 days before surgery, with no cessation or partial cessation).

### 2.1. ICH Assessment

**Figure 2:**
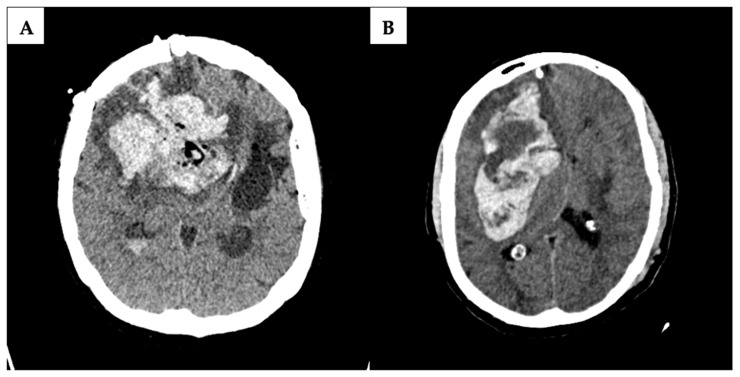
Postoperative computed tomography scans in a patient with postoperative intracranial hemorrhage. Examples of a severe intracerebral hemorrhage in the acetylsalicylic acid (ASA) group (**A**) and a massive intracerebral hemorrhage in the no ASA group (**B**). In both cases, a second operation was required.

All postoperative radiologic findings were reviewed for the presence of hemorrhage. The determination of blood volume was entrusted to radiology colleagues. Hemorrhages were systematically categorized as either hemorrhage in the tumor cavity; intracerebral hemorrhage; subarachnoid hemorrhage; or subdural hemorrhage. Significant postoperative hemorrhage was exclusively recorded for patients exhibiting substantial neurologic symptoms such as impaired consciousness due to increasing intracranial pressure and requiring surgical intervention due to space-occupying hemorrhage (Figure 2). Symptomatic neurologic deficits were precisely defined as focal neurologic deficit, headache, nausea, or a change in cognitive function.

### 2.2. Statistical Analysis

Categorical variables were reported as counts (%), whereas continuous variables were expressed as median (interquartile range [IQR]) values since all continuous variables in this study were nonnormally distributed, which was confirmed by the Kolmogorov–Smirnov test. The influence of categorical variables on pICH was analyzed by the χ^2^ or Fisher’s exact test, when numbers per field were <5. Differences in continuous variables between patients with and without pICH were compared in the Wilcoxon Mann–Whitney test. Factors significant in the univariate analysis were used as covariates in the multivariate analysis, which was performed using a logistic regression. In cases where variables exhibited substantial deviation from the normal distribution, a logarithmic transformation was applied. All statistical analyses were performed using the SAS University Edition software package 9.4 (SAS Institute, Inc., Cary, New York, USA) and SPSS for Windows version 18.0 (SPSS, Inc., Chicago, IL, USA). Two-sided *p* values <0.05 were considered statistically significant.

## 3. Results

During the study period, surgical procedures were performed on 365 patients presenting diverse types of metastases. The predominant metastatic type within this cohort was bronchial carcinoma (N = 148), followed by breast carcinoma (N = 58), cancer of unknown origin (N = 25), renal cell carcinoma (N = 20), and colon carcinoma (N = 19), among others. Notably, 21 patients underwent recurrent operations.

### 3.1. Incidence of ICH

**Figure 3:**
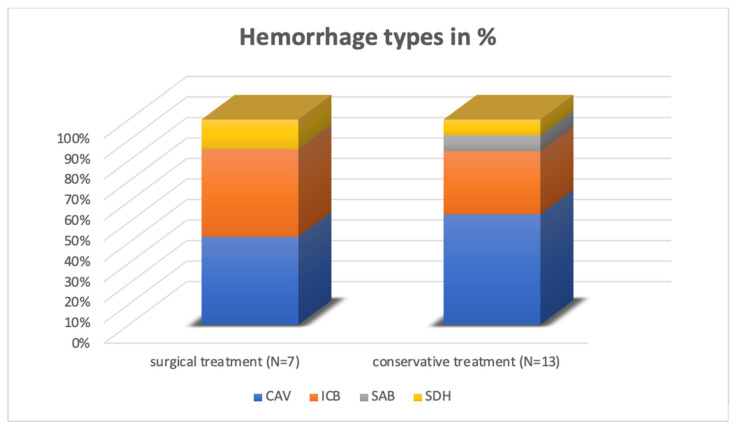
Distribution of hemorrhagic complications within the group of patients with intracranial metastases. Three patients with CAV were treated surgically and seven conservatively. Of the seven patients with ICH, only three underwent reoperation. Two patients had SDH, with only one patient requiring surgery due to postoperative hemorrhage. CAV, surgical cavity hemorrhage; ICH, intracerebral hemorrhage; SAH, subarachnoid hemorrhage; SDH, subdural hematoma.

A total of 20 patients (5.5%) experienced postoperative hemorrhage. Among them, 7 patients (1.9%) exhibited neurological symptoms or hemorrhages of a space-occupying nature necessitating reoperation and 13 (3.56%) patients had no symptoms despite post-operative hemorrhage, which is why reoperation was unnecessary. Table 1 and Figure 3 describe the frequency of hemorrhage within the various categories.

A majority of pICHs were identified as intracerebral (n = 7), with an additional prevalence observed in what is referred to as a resection cavity (n = 10), specifically post-tumor extirpation. Subdural hematoma (n = 2) and subarachnoid hemorrhage (n = 1) were comparatively less frequently identified through computed tomography. Figure 2 shows two examples where postoperative CT-imaging revealed space-occupying intracavital pICH requiring revision surgery.

Regarding ICH in relation to demographic characteristics and additional data, factors such as sex (*p* = 0.704), blood group (*p* = 0.230), smoking status (*p* = 0.131), age of the patient at the time of surgery (*p* = 0.977), BMI (*p* = 0.368), and comorbidities including multiple localization (*p* = 0.023), diabetes (*p* = 1.000), cardiovascular disease (*p* = 0.606), hypertension (*p* = 1.000), dyslipoproteinemia (*p* = 1.000), renal disease (*p* = 1.000), liver disease (*p* = 1.000), and chronic inflammation (*p* = 0.583) demonstrated no significant impact on the risk of postoperative hemorrhage.

In Table 2, relevant preoperative laboratory parameters, including coagulation factors, are presented. These data did not show any substantial influence on the risk of postoperative hemorrhage.

**Figure 4:**
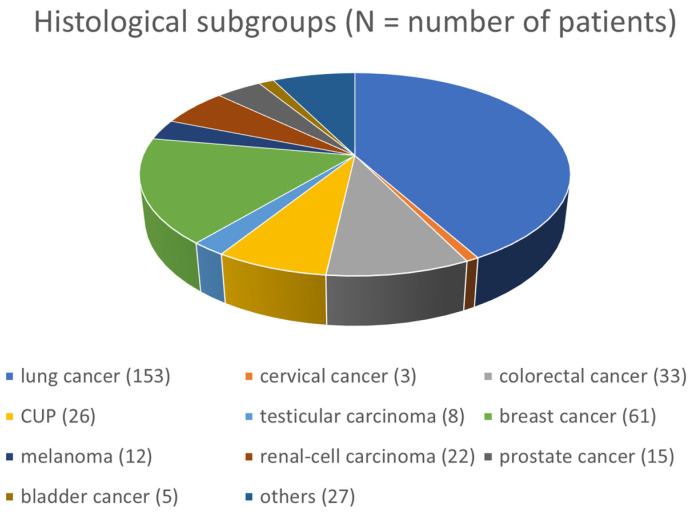
Distribution of patients according to histological subtype after known or newly diagnosed malignant disease.

Additionally, no significant associations between demographic data, comorbidities, tumor characteristics, and postoperative bleeding were identified. Figure 4 shows the distribution of patients according to histological subtype after known or newly diagnosed malignant disease.

### 3.2. Hemorrhage, Tumor Characteristics, and Laboratory Parameters

The tumor characteristics, encompassing histopathological metastatic type (*p* = 0.825); recurrent metastases (*p* = 1.000); metastatic location such as frontal, temporal, and skull base (*p* = 0.464); tumor site (*p* = 0.774); and surgical parameters such as operation duration (*p* = 0.217) and intraoperative blood loss (*p* = 0.143), underwent thorough evaluation. Among the laboratory parameters, only thrombin time exhibited significance in relation to the development of hemorrhage. Postoperative KPS (*p* < 0.001) and the GOS (*p* < 0.001) metrics exhibited significance in patients from both groups following hemorrhage. Moreover, there was a distinct trend towards an extended hospital stay post-hemorrhage in both patient groups (*p* = 0.038). Table 3 shows the laboratory parameters and intraoperative characteristics and Table 4 presents the outcome parameters.

**Figure 5:**
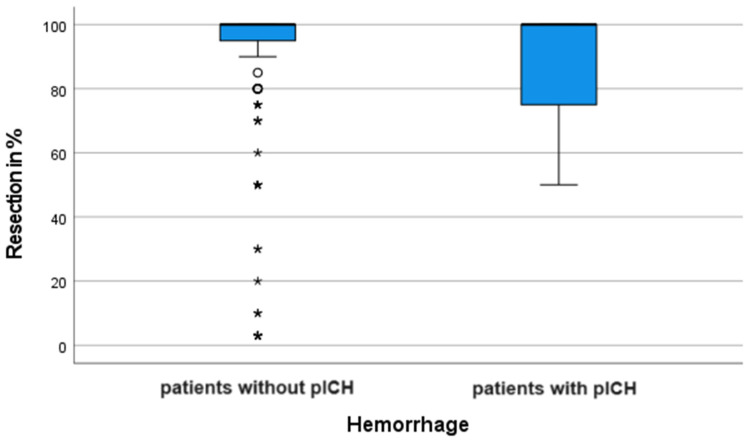
This box-and-whisker chart represents the 2nd and 3rd quartiles of tumor volume resection in the form of a rectangular (“box”) and key distribution ranges. Vertical lines extending from the top and bottom sides of the box plot the rest of the statistical distribution, and dots specify outliers within each patients’ group.

The evaluation of our data according to tumor volume resection suggested no difference between the patients with and without pICH. The median value of tumor volume resection among the patients with postoperative hemorrhage was 87.50% and that among the patients without hemorrhagic complication was 91.57% (*p* = 0.97). The following box chart in Figure 5 represents median values and key distribution ranges between both groups:

### 3.3. Patients with ASA Intake

Patients on ASA treatment were found to be predominantly male (*p* = 0.038) and exhibited a higher prevalence of smoking (*p* = 0.005), along with pre-existing conditions such as diabetes (*p* = 0.069), heart disease (*p* < 0.001), dyslipoproteinemia (*p* = 0.022), liver disease (*p* = 0.013), chronic inflammation (*p* = 0.005), and hypertension (*p* < 0.001) (Table 5). Furthermore, patients older than 61.6 years of age were more frequently administered ASA as a premedication (*p* < 0.001). A total of 72 patients (19.7%) had a history of ASA at the time of surgery, and among them, 58 (15.9%) were classified under the continued ASA use group.

Patients with continued ASA use (ASA impact) did not exhibit a significantly elevated incidence of postoperative hemorrhage requiring surgery when compared to the group with no ASA impact (*p* = 0.120). The occurrence of hemorrhage did not manifest more frequently in the group with continued ASA use compared to the group with discontinued ASA intake before the operation (*p* = 0.293).

### 3.4. VTE and ASA Intake

All patients were provided with standard-issue compression stockings following the surgical procedure and were encouraged to mobilize themselves on the first postoperative day. Even patients who had sustained paralysis and were unable to undergo full mobilization were offered professional physiotherapy with movement exercises in bed in the postoperative period.

In the group with no ASA impact, 11 of 307 patients (3.6%) developed pulmonary artery embolism (PE), while 2 of 58 patients (3.4%) in the ASA impact group experienced this outcome. No significant difference was observed between the two groups (*p* = 1.000) In our cohort, six patients suffered deep venous thrombosis (DVT), with only one of them being on ASA medication. This patient was diagnosed with both PE and DVT.

### 3.5. Complications According to Ibanez Classification

Table 6 illustrates the frequency of complications in both groups, utilizing the Ibanez classification for comparison. It helps to gain a general overview of the postoperative adverse events. Among the patients with pICH, the most common were complications grade III a/b (71.4%), and among those without pICH, grade I a/b (12.5%). Two of the seven patients who experienced pICH died in the course of hospital treatment. There was no substantial difference between both groups, whether with or without ASA. A significant correlation between severe complications was observed, especially post-operative hemorrhage and mortality (*p* = 0.001). The fact that patients with Ibanez grade IV complications exhibited a notably greater association within the group of ASA impact patients (*p* = 0.038) correlated with the higher incidence of multiple comorbidities in those patients. It could be assumed that patients who were not able to cease ASA intake were mostly at an elevated risk of unfavorable surgical outcome in the event of a complication occurring after surgery.

## 4. Discussion

Cerebral metastases are the most common type of intracranial tumor [1,2]. The importance of ASA use for primary and secondary prophylaxis increases with age [7,8]. Certain metastases carry a heightened risk for hemorrhage, but the incidence of pICH varies significantly among different studies, contingent upon the specific definition employed [1,24]. The decision to administer a therapeutic dose of aspirin preoperatively to patients with metastases is difficult due to the potentially fatal consequences of ICH [7,8]. Acute hemorrhagic and thromboembolic outcomes following intracranial surgery for brain tumors can have dire consequences for patients. Analyzing the factors contributing to the mentioned complications is paramount for saving patients’ lives. However, the correlation of ASA use with pre- and intraoperative conditions after tumor resection has rarely been investigated [9].

In our patient population, the occurrence of clinically relevant pICH was 1.9%, which is within the reported incidence range described in the literature (0.8–6.9%) [8,25,26]. There was no significant difference in the incidence of postoperative hemorrhage in patients taking ASA preoperatively. This finding aligns with observations in neurosurgical studies that focused on ASA and skull surgeries [9,10,11]. While some authors have reported increased incidents of ICH events associated with ASA [27,28], other researchers have found no significant impact of ASA on intracranial rebleeding [5,29]. In a recent study by Rahman et al. involving patients who underwent craniotomies for intracranial tumors, the analysis of those taking ASA compared to the control group showed no significant differences with regard to the occurrence of pICH or thromboembolic systemic complications [5]. This finding suggests that patients can safely continue using ASA for primary and secondary prophylaxis before surgery.

An analysis of the patient demographic characteristics and additional data showed no significant differences between the two groups. However, patients with multiple pre-existing illnesses experienced hemorrhage more frequently than those without such conditions. This is similar to what has been described by Donato et al. and Mantia et al. [4,30], although these studies examined hemorrhage frequency and the administration of enoxaparin and not ASA.

Some authors have noted that tumors are a common cause of postoperative hemorrhage during intracranial procedures [31,32] and the degree of lesion vascularization may play a role in the development of pICH. The increased risk of secondary bleeding in hypervascularized processes may be attributed to pathological and more vulnerable vessel walls, as well as the activity of tumor-specific enzymes and growth factors. These processes particularly involve lesions that have rapidly infiltrated the brain parenchyma, such as metastases. Notably, if a tumor is only partially removed, the risk of secondary bleeding significantly rises [31,32]. In such cases, the brain loses local elasticity, and after tumor removal, a resection cavity often persists. This cavity can gradually fill with blood over the next few hours or days due to small capillary and/or venous bleeding [25,33]. The resection percentage of the tumor in our study revealed no significant difference between patients with and without pICH; however, a tendency, as in the above-mentioned studies, was observed.

There is considerable variability in the reported data on VTE in brain tumor patients following craniotomy. For instance, one study indicated a 3.0% incidence of VTE in brain tumor patients undergoing craniotomy [34], which is similar to our findings. According to the current literature, VTE occurs more frequently in patients with malignant gliomas, with an annual risk of up to 18%, and a cumulative lifetime risk of approximately 30% [35,36]. In contrast, another study [12] reported a VTE incidence of 13.7%, where asymptomatic patients were also examined for the presence of VTE.

In our cohort, the incidence of VTE was comparable in both groups and consistent with findings in other studies [37,38] involving craniotomies. It is important to note that only symptomatic patients were further investigated for thrombosis. All patients were provided with compression stockings and were encouraged to mobilize immediately following the first postoperative day. Even patients with paresis, who could not be fully mobilized, received professional physiotherapy with bedside range of motion exercises after surgery. In this context, no significant difference was observed between the ASA and non-ASA groups.

Among the tumor characteristics, histology, and laboratory parameters, only thrombin time emerged as a significant factor for the development of postoperative bleeding. The analysis of patient demographics and additional data did not show any significant differences. Patients with multiple pre-existing illnesses experienced bleeding more frequently than those without such conditions. This is in contrast to Smith et al., who observed that, among the demographic parameters, gender and age were significant factors for the development of VTE [12]. We found no statistical evidence to validate the hypothesis [39] that a platelet count below 150/nl significantly increases the risk of pICH, primarily due to the low number of patients with thrombocytopenia in our study. For example, statistical calculations pertaining to pICH were not deemed meaningful for a small subset of patients with pICH and abnormal partial thromboplastin time, international normalized ratio, or Quick values.

## 5. Conclusions

Future research should investigate thromboprophylactic therapy, evaluate the individual risk profiles of patients, and screen for asymptomatic events as well as the occurrence of VTE and/or ICH. These inquiries are essential for advancing our understanding and refining strategies for managing postoperative complications in patients undergoing surgery for intracranial metastases. A limitation of these data is the retrospective nature of the study design. Platelet function tests were not conducted routinely before surgery, thus limiting the generalizability of our findings. Prospective studies should be used to validate these results with the multiplate^®^ test and the platelet function test in patients taking ASA preoperatively. Finally, another limitation was the study size and inclusion of a low number of patients with thrombocytopenia.

## Figures and Tables

**Table 1:** Classification of patients according to acetylsalicylic acid (ASA) impact and hemorrhagic complications leading to revision surgery.

				Hemorrhage with Operation	No Hemorrhage	*p*-Value
				N (%)	N (%)	
**ASA Impact**	**Yes** **No**	**∑ (%)**	**∑** 58 (15.9) 307 (84.1) 365	2 (28.6) 5 (71.4) 7 (100.0)	56 (15.1) 302 (84.9) 358 (100.0)	**0.120**

**Bold** font represents statistically significant results (*p* < 0.05).

**Table 2:** Patient distribution according to demographic data as well as comorbidities and tumor characteristics and their correlation to hemorrhagic complications. None of these parameters showed significant influence on postoperative bleeding.

Parameters		Hemorrhage with Operation	No Hemorrhage	*p*-Value
		N (%)	N (%)	
Demographic data				
Sex	Female	2 (28.6)	144 (41.7)	0.704
	Male	5 (71.4)	201 (58.3)	
Age (mean ± SD, years)		63.12 ± 9.03	62.71 ± 11.27	0.977
Blood group	A	1 (16.7)	149 (44.1)	0.230
	B	0 (0.0)	39 (11.5)	
	AB	0 (0.0)	20 (5.9)	
	O	5 (83.3)	130 (38.5)	
Smoker	Yes	1 (14.3)	157 (46.3)	0.131
	No	6 (85.7)	182 (53.7)	
BMI (mean ± SD)		26.86 ± 2.14	26.41 ± 5.60	0.368
Comorbidities				
Diabetes	Yes	1 (14.3)	61 (17.7)	1.000
	No	6 (85.7)	284 (82.3)	
Hypertension	Yes	3 (42.9)	172 (49.9)	1.000
	No	4 (57.1)	173 (50.1)	
Cardiovascular diseases	Yes	0 (0.0)	59 (17.1)	0.606
	No	7 (100.0)	286 (82.9)	
Coagulopathy *	Yes	0 (0.0)	5 (1.4)	1.000
	No	7 (100.0)	340 (98.6)	
Tumor characteristics				
Localization	frontal	1 (14.3)	80 (23.2)	0.464
	temporal	1 (14.3)	43 (12.5)	
	parietal	1 (14.3)	88 (25.5)	
	occipital	0 (0.0)	50 (14.5)	
	cerebellar	4 (57.1)	72 (20.9)	
	brainstem	0 (0.0)	1 (0.3)	
	intra-/suprasellar	0 (0.0)	4 (1.2)	
	skull base	0 (0.0)	2 (0.6)	
Side of tumor	left	3 (42.9)	169 (49.0)	0.774
	right	4 (57.1)	166 (48.1)	
	middle	0 (0.0)	10 (2.9)	

BMI, body mass index; SD, standard deviation. * Coagulopathy of insufficient clotting type.

**Table 3:** Laboratory parameters in relation to post-surgical outcomes. Among the laboratory parameters Thrombin time showed significant effect on postoperative hemorrhage risk. None of the other parameters exhibited a relevant risk-factor for the development of pICH.

		Hemorrhage with Operation	No Hemorrhage	*p*-Value
		N (%) Mean ± SD Median (1.q; 3.q)	N (%) Mean ± SD Median (1.q; 3.q)	
Laboratory parameters				
	WBC count [Gpt/l]	7 (2)	345 (98)	0.253
		14.11 ± 7.47	10.95 ± 4.62	
		11.60	10.10	
	Platelet count [Gpt/l]	7 (2)	345 (98)	0.753
		286.57 ± 96.40	283.57 ± 104.33	
		334.0	275.0	
	Partial thromboplastin time [s]	7 (2)	345 (98)	0.320
		26.09 ± 4.88	27.13 ± 4.33	
		24.8	26.9	
	INR	7 (2)	345 (98)	0.283
		1.08 ± 0.23	1.00 ± 0.07	
		1.00	0.98	
	Thrombin time [s]	7 (2)	345 (98)	**<0.001**
		19.21 ± 2.97	16.64 ± 2.01	
		18.4	16.3	
Intraoperative characteristics				
	Blood loss [mL]	7 (2)	345 (98)	0.143
		415.71 ± 266	329.45 ± 324	
		300.0	250.0	
	Duration of operation [min]	7 (2)	345 (98)	0.217
		199.0 ± 48.7	178.1 ± 68.0	
		216.0	169.0	

**Bold** font represents statistically significant results (*p* < 0.05). 1.q, first quartile; 3.q, third quartile; Gpt, gigaparticles; INR, international normalized ratio; SD, standard deviation.

**Table 4:** Outcome parameters (hospital stay duration, together with KPS and GOS), showing a significant correlation with clinically relevant postoperative hemorrhage.

		Hemorrhage with Operation	No Hemorrhage	*p*-Value
		N (%) Mean ± SD Median (1.q; 3.q)	N (%) Mean ± SD Median (1.q; 3.q)	
Intraoperative characteristics				
	Hospital stay [days]	7 (2)	345 (98)	**0.038**
		19.86 ± 7.1	15.08 ± 7.6	
		21.0	13.0	
	Karnofsky Performance Score	7 (2)	345 (98)	**<0.001**
		32.86 ± 25.64	67.13 ± 18.42	
		30.0	70.0	
	Glasgow Outcome Scale	7 (2)	345 (98)	**<0.001**
		2.43 ± 1.2	4.20 ± 0.96	
		2.0	4.0	

**Bold** font represents statistically significant results (*p* < 0.05). 1.q, first quartile; 3.q, third quartile; SD, standard deviation.

**Table 5:** Demographics of acetylsalicylic acid (ASA) groups. Patients in the ASA impact group were older than those in the no ASA impact group; had a poorer ASA operative score; and suffered more frequently from concomitant diseases such as arterial hypertension, cardiovascular diseases, and chronic inflammatory disorders.

	Parameters		ASA Preoperatively		*p*-Value
			No ASA Impact N Total = 307 (100%)	ASA Impact N Total = 58 (100%)	
Demographic data					
	Sex	Female	135 (44.0)	17 (29.3)	**0.038**
		Male	172 (66.0)	41 (70.7)	
	Age (Mean ± SD, years)		61.7 ± 11.3	68.6 ± 8.15	**<0.001**
	BMI (Mean ± SD)		26.41 ± 5.48	26.68 ± 5.55	0.735
	ASA score	I	4 (1.3)	0 (0.0)	**<0.001**
		II	147 (47.9)	12 (20.7)	
		III	148 (48.2)	45 (77.6)	
		IV	8 (2.6)	1 (1.7)	
	Smoker	Yes	127 (42.2)	36 (62.1)	**0.005**
		No	174 (57.8)	22 (37.9)	
Comorbidities					
	Diabetes	Yes	49 (16.0)	15 (25.9)	0.069
		No	258 (84.0)	43 (74.1)	
	Hypertension	Yes	138 (45.0)	42 (72.4)	**<0.001**
		No	169 (55.0)	16 (27.6)	
	Cardiovascular diseases	Yes	34 (11.1)	28 (48.3)	**<0.001**
		No	273 (88.9)	30 (51.7)	
	Coagulopathy *	Yes	5 (1.6)	1 (1.7)	1.000
		No	302 (98.4)	57 (98.3)	
	Chronic inflammation	Yes	29 (9.4)	13 (22.4)	**0.005**
		No	278 (90.6)	45 (77.6)	
Operative characteristics					
	Duration of operation [Mean ± SD in min]		178.14 ± 68.0	196.93 ± 100.4	0.221
	Blood loss [Mean ± SD in ml]		330.52 ± 322.51	420.69 ± 533.86	0.298
	Duration of hospital stay [Mean in days]		15.26	15.41	0.702
Outcome parameters	Karnofsky Performance Score [%]		66.74	63.62	0.853
	Glasgow Outcome Scale (1–5)		4.18	4.02	0.953

**Bold** font represents statistically significant results (*p* < 0.05). BMI, body mass index; SD, standard deviation. * Coagulopathy of insufficient clotting type.

**Table 6:** Surgical and medical complications after brain metastasis surgery grouped according to occurrence of complications within the postoperative period. There is a strong correlation between the occurrence of postoperative hemorrhage and mortality (Ibanez IV) in both surgical and medical classification subgroups.

According to Ibanez’s Classification:		Hemorrhage N (%)	No Hemorrhage N (%)	*p*-Value *
Surgical complication:	None	0 (0.0)	270 (78.3)	**<0.001**
END/Seizure	Ia/Ib	0 (0.0)	43 (12.5)	
CSF leaks/Wound infection	IIa/IIb	0 (0.0)	15 (4.3)	
Hemorrhage/Cerebral edema	IIIa/IIIb	5 (71.4)	6 (1.7)	
Death	IVa/IVb	2 (28.6)	11 (3.2)	
	∑	7 (100.0)	345 (100.0)	
Medical complications:	None	3 (42.9)	297 (86.1)	**<0.010**
Lung infection/UTI	Ia/Ib	2 (28.6)	14 (4.1)	
DVT or PE/Pneumothorax	IIa/IIb	0 (0.0)	20 (5.8)	
Lung failure/Reintubation	IIIa/IIIb	0 (0.0)	3 (0.9)	
Death	IVa/IVb	2 (28.6)	11 (3.1)	
	∑	7 (100.0)	345 (100.0)	

* Examination with Fisher–Freeman–Halton Test. **Bold** font represents statistically significant results (*p* < 0.05). Complications are classified as mild (Ibanez I), moderate (Ibanez II), and severe (Ibanez III and IV). END, early neurological deterioration; CSF, cerebrospinal fluid; UTI, urinary tract infection; DVT, deep venous thrombosis; PE, pulmonary embolism.

## Data Availability

The datasets obtained and analyzed during the current study are available from the corresponding author on reasonable request.
